# Reconstruction and Resilience in Rwandan Education Programming: A News Media Review

**DOI:** 10.1177/15423166231179235

**Published:** 2023-06-01

**Authors:** Brandon A. Dickson

**Affiliations:** Balsillie School of International Affairs, 8430 University of Waterloo, Waterloo, ON, USA

**Keywords:** peace education, Rwanda, resilience, post-conflict education, global governance

## Abstract

This study evaluates news portrayals of resilience in the newly renewed Education for Sustainable Peace in Rwanda initiative. There remains a gap in understanding about how Rwanda's education approaches are portrayed and disseminated to the public by news media. This research uses an inductive coding analysis and layers Entman's framing theory to evaluate new media portrayals of resiliency in the reporting of domestic and international media outlets. This research demonstrates that there has been little media attention paid to Rwanda's education system outside of Rwanda, despite the newly revised programming. This research also finds that in media sources which do discuss Rwanda's education system, portrayals of approaches to resilience that have the potential for inclusivity are far more common than approaches which are top-down and exclusive. These findings serve to contribute to literature in both the context of Rwanda's place in global governance and the broader discussions of educational resiliency.

## Introduction

Following Rwanda's 1994 genocide of 800,000 ethnic Tutsi, moderate Hutu and Twa (Uvin, 1997), there has been massive international intervention, specifically in Rwanda's education system, trying to build resilient citizens who can create resilient peace. To implement peace education focused on developing resilience against genocide, the Education for Sustainable Peace in Rwanda (ESPR) initiative was launched by British NGO Aegis Trust in 2017 and was renewed in 2020 (Basabose & Habyarimana, 2019).

The education system in Rwanda, like much of the international aid they receive, often receives very little scrutiny internationally, due to their donor darling status (Reyntjens, 2011). While there is much research on the success of Rwanda's various education initiatives and the development of a precarious peace education (Sundberg, 2016; Thomson, 2011; 2016), there has been much less research on the portrayal of the new education initiatives both in Rwanda and internationally. A key avenue of which is the portrayl of programming by news media (Fryberg et al., 2012), which is especially important given that media coverage of education reform is often political and contributes to shaping public perception (Kelly, 2011).

This research explores media portrayals of genocide education programming in Rwanda through the following question: How are international best practices for developing post-conflict resilience in education for the new Rwandan peace education programming portrayed in the reporting of domestic and international media outlets?

In this study, I begin by exploring the broader context of the resilience literature with a focus on resilience in education. I then use the literature on peace education to develop a series of best practice codes which form the basis for evaluation of the news sources in their portrayal of ESPR. Using Entman's framing theory (Entman, 1993, 2007, 2010; Entman & Usher, 2018), news sources are also sorted according to one of his four frames.

This research demonstrates that there has been very little media attention paid to Rwanda’s education system outside of Rwanda, despite the newly revised education programming. This research also finds that in media sources which do discuss Rwanda's education system, portrayals of approaches to resilience that have the potential for inclusivity are far more common than mention of approaches which are top-down and exclusive. These findings serve to contribute to literature on both Rwanda's peace education and educational resiliency as a whole.

## Literature Review

### Peace Education

Peace education goes by many other names, but at its core, peace education programs main goals include, “explaining the roots of violence and teaching alternatives to violence given that conflict is omnipresent” (Harris, 2004; p. 5). In some cases, this education is focused on the prevention of conflict, and in others, it is focused on the sustainability of peace (Harris & Morrison, 2012).

Often times, peace education is about building in resilience as a framework for other goals, that is bouncing back from conflict to achieve social success, such as mutual acceptance, and economic success (Bekerman & Zembylas, 2011). Many peace education programs are built into peace agreements facilitated by international actors (Dunlop & King, 2021), and are operated by or at least funded in-part by international groups (Autesserre, 2012) who do not necessarily have a background in peace education (Burde, 2014). This is particularly relevant in the case of Rwanda where much of the peace education programming is being implemented by British NGO Aegis Trust.

Peace education often begins with history education, which serves contradictory roles, the training of critical thinking, and the teaching of nationalist histories (Skårås, 2021). Recent research has found that history in peace education which emphasizes a single narrative serves to cultivate further intolerance through ethno-nationalist narratives (King & Samii, 2020; Skårås, 2021). Contemporary international examples of this include the banning of textbooks which tell both the Israeli and Palestinian view in Israel (Lässig, 2013), and the growing ‘apology fatigue’ in the Japanese context where joint history projects with China and South Korea have been abandoned in favour of nationalist histories (Fukuoka, 2018) In response to this, many peace education programs have reshaped themselves away from the actual conflict and as citizenship education programmes which aim to create informed citizens through moral and social and emotional education (Hymel & Darwich, 2018). Oftentimes such an education programs can avoid controversial issues including history and ethnicity in favour of promoting international citizenship and recognition (Quaynor, 2012), an approach which as was found with holocaust education, can limit the transferability of such skills to students actual lives (Karn, 2012). Instead, international examples such as the North Ireland context (Reynolds & Blair, 2018) and the South African context (Bennett, 2012) note the importance of teaching directly about the troubling history and leaving space for debate and interpretation.

In this context then, Rwanda stands out as a unique case study. Rwanda was, early on, the subject of immense international intervention in education following the genocide. Under current President Paul Kagame, Rwanda has been touted as the model for reconstruction and international intervention. Kagame has done away with ethnic indicators, Tutsi, Hutu and Twa, and has pushed citizens to rally behind being simply Rwandan (Moran & Samso, 2019). Since the mid-2000s, Rwanda has followed the official narrative of the ‘genocide against the Tutsi’ (Prunier, 2009). This ‘Tutsification of the genocide’, has resulted in a much stricter national narrative, which not only excludes Hutu and Twa victims, but also silences the voices of those impacted by RPF violence.

In Rwandan peace education, there was a 15-year moratorium period on the teaching of Rwandan history and the genocide (Skårås, 2021). When history teaching came back, it was focused on reconciliation, holding past governments responsible and bestowing national forgiveness (Zorbas, 2009). Part of Rwanda's top-down education programming was the focus on Rwandan-ness to achieve unity and accelerate reconciliation through the narrative of ‘One Rwanda’ (Thomson, 2011). Given Rwanda's history with education as a driving factor in the genocide, the ‘add education and stir’ approach was insufficient for solving conflict. Especially as Rwanda's ethnic history was based on European constructions of race, the educational challenge is all the more daunting (King, 2005).

Much research has been conducted on Rwanda's peace education approach. Rwanda's peace education approach is notable in that it is not limited exclusively to schools, but expands to genocide memorials, which are sanctioned by the state (Ibreck, 2010). They serve as the centre piece for much of the history teaching beyond school age children (Sodaro, 2011) and have received a great deal of international funding and support aimed at developing an international presence (Ibreck, 2013). Some of the early attempts at peace education occurred in what was called Itorero, a citizenship education program which was part of Rwanda's vision 2020 initiative and had community leaders lead weekly seminars to discuss Rwandan history, values and taboos in order to train “model citizens” and make people engage with the state and their neighbours (Sundberg, 2016). While some have noted that this program was successful in changing Rwandan's self-image to being a nation focused on togetherness (Dahlmanns, 2015), others have noted that this singular focus on civic education removes citizens’ ability for healthy dissent (Nzahabwanayo, 2018).

In an attempt to consolidate and expand much of this education programming, Rwanda's current peace education initiative, Education for Sustainable Peace in Rwanda (ESPR) was created as an extension of Rwanda's earlier Peace and Values Education. The role of ESPR is broadly to train individuals who are resilient against genocide. The ESPR programming saw an updated curriculum implemented which infused peace teaching across the curriculum and saw both elementary and teachers receive training and model lesson plans to implement peace education in their schools (Basabose & Habyarimana, 2019). ESPR's main goals include the training of teachers in peace education, the teaching of peace education to students in classrooms, the use of community peace schools in Karongi and Kigali to host peace programs for the general public, and to train parents in the teaching of peace education principles (Russell, 2018). Rather than replacing past peace education training, ESPR was brought in to serve as the umbrella over which peace education could be organized, and as such, past research on peace education programming includes focus on various aspects of the ESPR program. This project, which was renewed in 2020 (Aegis Trust, 2020), serves as a partnership between Aegis Trust, and the Rwandan Ministry of Education, and is also funded by both the Governments of Sweden and the United Kingdom (Aegis Trust, 2017).

Recent research on the approaches of new education programming in Rwanda has noted that their programs are often resistant to recognizing differences in identity (King & Samii, 2020) and serves as a strategic tool to continue to write the governments narrative (King, 2017). Others have noted that secondary curriculums are often contradictory to students own experiences and faces limits in implementation (Basabose & Habyarimana, 2019). These provide limitations to the success of the programming to create resilient citizens in line with international best practices. Despite the broad recognition of the challenges facing the Rwandan education systems in peace education and its implementation of effective resilience, there has been a lack of research on how coverage of this peace education has portrayed the program in Rwanda and internationally.

Despite the broad recognition of the challenges facing the Rwandan education systems in peace education and its implementation of effective resilience, there has been a lack of research on how coverage of this peace education has portrayed the program in Rwanda and internationally.

With all of this in mind, it is important to note that Rwanda's broader peace education initiatives, including ESPR while received mixed reviews from scholars still have broad international support, which shows a relative contentedness in carrying on with this education strategy as adopted by Aegis in Rwanda. Understanding the media portrayals of such an approach to the development of resiliency serves to support an understanding not only of how ESPR is perceived within and beyond Rwanda and the ways in which this understanding might lead to perceptions of the efficacy of such programming (Fryberg et al., 2012; Kelly, 2011).

## Conceptual Framework

### Resilience in Education

As noted above, resilience and peace education are inherently intertwined, with the purpose of peace education programming being to train students to be resilient against conflict. Resilience has been conceptualized as everything from an ideology to a governing rationality, and a programme to a buzzword (Anderson, 2015), but prioritizes individual agency and liberal international peace (Chandler, 2013; Joseph, 2013), especially in the context of peace education where resilient individuals are necessary to create a peaceful society.

Particularly in education, resilience seeks to promote academic success and well-being in both students and educators as part of the broader goal (Brewer et al., 2019). Traditional education however may not provide students with skills to succeed beyond the classroom. Resilience, as a part of peace education programming can fill this void (Goertzen & Whitaker, 2015). Resilience education is often focused away from individual student success and emphasizes the return to normal for all, with a focus on adaptation to changes. As such, focus on student wellbeing is secondary to resilience development (Brewer et al., 2019; Hartley, 2013).

This research bases itself in the notion that resilience is a policy approach targetted at the individualization of preparation and response to disaster, targetting a return to normal for the majority which can be either positive or negative or both depending on the individual or community (Bonds, 2018; Chandler, 2013; Gillham et al., 2013; Gu, 2018; Rogers, 2013). Especially in the context of peace education programming, there is not one best way to promote resilience, but rather there are certain approaches which may be more beneficial for different groups of people. As such, this research now situates the Rwandan context beside broader understandings of resilience in peace education to understand how positive and negative resilience would look in the context of this study.

Resiliency in the context of Rwandan peace education is more likely to occur when multiple stakeholders have a say in the rebuilding of their community (Rogers, 2013; Ungar, 2018). In the implementation of Rwandan peace education there are many approaches which may demonstrate inclusive resilience. This can include programs which offer delivery differentiation to meet varying needs of the community and also supporting programming which moves beyond traditional classrooms (Salomon, 2011). Further, the inclusion and recognition of difference in ethnicities provide the groundwork for meaningful long-term resilience (Skårås, 2021). Lastly, the promotion of skills such as critical thinking serve as the pillar for which to build back resilience (Krasny et al., 2009).

In contrast, when resilience is implemented in a top-down fashion with an emphasis on the opinions of experts and a return to the status quo, the impacts of education can be harmful. In the Rwandan context, the programming being run by an international NGO in Aegis Trust, does not necessitate a dangerous exclusive peace education resilience approach, but may devolve into this. For example, teachings such as the removal of historical complexities in favour of single narratives which all are expected to adopt (Skårås, 2021) as well as a nationalist focus can promote exclusionary resiliency (Zembylas, 2016). Similarly, programming which devolves into simplified lessons of citizenship, rather than instruction on history, position the nation for conflict (Quaynor, 2012). Recognizing resilience as a cornerstone of successful peace education, exploring the ways in which the ESPR programs’ approach to resilience development in Rwanda are understood and portrayed in media is important to understand how public perception is shaped on the success of ESPR (Fryberg et al., 2012; Kelly, 2011).

### Framing Theory

This research bases itself on Entman's framing theory (Entman, 1993; 2007; 2010, Entman & Usher, 2018), which notes that framing in news is to make some aspects more salient to the reader. Entman notes that frames define problems, diagnose causes, suggest remedies, and make moral judgements on the problem being reported. This framing is, “manifested by the presence or absence of certain keywords, stock phrases… that provide thematically reinforcing clusters of facts or judgments” (Entman, 1993; p. 52). It is important to note the way that the reader receive this information may not necessarily be the same as the way it is presented. Nevertheless, frames determine whether and how people understand a problem, which then influences how they will act (Entman, 1993). While social media has changed how people consume news media, the core idea of framing has remained consistent (Entman & Usher, 2018). Framing theory is often used in comparisons of international news to contextualize varying perspectives and to understand how people consume an issue (Fryberg et al., 2012).

## Methodology

### Procedures

#### Document Collection

A search for newspaper articles was conducted in May 2022. Key terms are as follows:

- Rwanda peace education
- Education for Sustainable Peace in Rwanda
- Rwanda resilience education
- Rwanda genocide education
- Rwanda values education
- Rwanda Aegis Trust education

These terms were searched for from the following news sources sorted by region. Rwandan sources were expected to make up most of the data for this research due to lack of coverage on education in the area. The focus of international newspaper selection was on British and American sources due to their prominent news coverage of Africa. The Rwandan sources are the two main English publications in Rwanda and were chosen for this reason. The international sources represent well known English international publications which have an international focus and have historically provided coverage on the African continent and Rwanda. These sources, therefore, provide a substantial sample of international coverage to draw on for analysis. Many of these newspapers’ archives also serve as databases compiling or republishing other sources beyond just their outlet:

*Rwandan Pro-Government Publications:*

- The New Times
- Kigali Times

*African Regional English Publication:*

- All Africa

*International News Sources:*

- Canadian Broadcasting Corporation (Canada)
- The New York Times (United States)
- The Washington Post (United States)
- Al Jazeera (Qatar)
- British Broadcasting Corporation News (United Kingdom)
- The Independent (United Kingdom)

and databases containing newspapers:

- The Genocide Archives of Rwanda
- Google News

Additional selection criteria included that the articles had to be available in English, due to the authors’ language limitations. While this excluded sources in Kinyrwanda or French, the broad uptake of English in publishing in Rwanda provided significant data for analysis. Articles also had to be published post-2014, the year development of a successor peace education program, which became ESPR, began. All articles on education broadly were collected for further consideration. In total, 35 articles were collected. After initial review, eight articles were removed from this study, due to their focus on non-peace education, leaving 27 articles for analysis. While many news outlets included in this search had coverage on ESPR, they almost exclusively republished articles from The New Times or All Africa, which led to most articles being from these two sources, 5 articles from All Africa and 17 from The New Times. The remainder were from international sources. While this small sample size does not allow for the same sweeping claims as other framing analyses, it reveals one of the main findings of this research discussed later, a broad lack of interest and coverage on peace education in Rwanda.

#### Data Source Review

Based on the readings from post-conflict education, this research adopts a thematic coding analysis to evaluate media portrayals of Rwanda's post-conflict education programming (Braun & Clarke, 2012). I layer a deductive coding approach to my thematic analysis, meaning codes were derived from the literature in advances so that they could then be mapped onto news articles (Braun & Clarke, 2012; p. 58). Codes were developed which embody approaches to resilience in peace education based on their prominence in the literature. While not thought of as an exhaustive list, these terms stood out as the most common, and cover a substantial portion of the necessities for post-conflict education. Table 1 below gives a definition for each code and a reference to the literature which resulted in the code.

**Table 1.** Code List with Definitions and Citations.

Code	Description
Capital Interests	The use of resilience solely to advance economic benefit for an individual, organization or society (Bonds, 2018; Bulley, 2013)
Citizenship Education	The use of education programs favouring personal growth or moral teaching avoiding controversy (Bevington et al., 2020; Hymel & Darwich, 2018)
Combatting Denial	The focus on preventing genocide denial as part of teaching (McLean Hilker, 2011)
Community	The focus on supporting community development and outreach beyond the school (Sundberg, 2016)
Critical Thinking	The promotion of critical thinking skills (Krasny et al., 2009)
Debate	The creation of room for difference in opinion in how education ought to be implemented or received by various groups (Rogers, 2013)
Difference	The allowance for individual stories and experiences to be told (Skårås, 2021)
Ethnicity	The discussion and allowing of ethnic identities as part of peace education training (King & Samii, 2020)
Preventing Genocide	The use of education to prevent future conflict (see for example: Harris & Morrison, 2012)
Nationalism	The focus or promotion of the national good or vision (Zembylas, 2016)
Return to normal	The emphasis on getting students back to their pre-trauma state (Brewer et al., 2019; Hartley, 2013)
Single narrative	The focus on one government endorsed narrative above individual experience (Skårås, 2021; Zembylas, 2016)
Top-down	The development of education materials by government officials to be used by educators (Bonds, 2018; King & Samii, 2020; Rogers, 2013)
Tolerance	The recognition of varying experiences and multiple perspectives as part of the education programming (Bonds, 2018; Ungar, 2018)

References to each code were deemed to occur if the newspaper article mentioned any approach or thought which directly or indirectly invoked the definition of a code. That is, the code analysis cannot be entirely divorced from their context, and required an interpretation so that terminology which embodied a specific code was incorporated correctly. For example, the notion of ‘One Rwanda’ is inherently a nationalist ideology included in Rwandan education and was therefore coded as nationalism. This aligns with the latent approach to thematic analysis (Braun & Clarke, 2006).

Based on the above data collection, analysis took place in three stages. In the first, publications were reviewed to determine the geographic interest in Rwanda's education system. This step allowed for an exploration of which areas of the world reported on Rwanda's post-genocide education. The second phase involved the reading of each newspaper and determining where instances of the above-mentioned codes occurred in each text. This developed an understanding of which approach to resilience in peace education was reported in Rwanda's education system. The third stage involved the recoding of each code to one, or more, of Entman's four approaches to the framing media. A specific resilience code could be coded to multiple frames, and therefore more frames exist than codes.

To ensure accuracy, an intrarater agreement test was conducted, where the author coded each article one month apart to check for consistency in coding. The codes were identical 90.4% of the time, with no new codes added and one code removed during stage two and 94.2% for stage three. Inconsistencies in codes were rectified. In total, the 13 codes from Table 1 were found a total of 51 times and included for analysis. Two articles did not receive a code.

## Results and Discussion

For the purpose of clarification, Table 2 below gives an example for each code for illustrative purposes, which forms the basis for the deductive analysis of the newspaper articles. Based on the code development and literature connections noted in Table 1 above, examples are presented here to show what each code might look like in the context of the various news sources along with a justification. For example, based on the work of Bevington et al. (2020) and Hymel and Darwich (2018), a code of citizenship education is presented in the context of resilience education, with a focus on personal growth, rather than actual teaching about the conflict. The purpose of this table is not to say any quote is inherently positive or negative, but rather to set the stage for later discussion.

**Table 2.** Code Example and Justification.

Code	Example	Justification
Capital Interests	n/a	n/a
Citizenship Education	Just as people were taught to hate one another, we can also teach people to love one another. When we teach peace, we develop a generation of peacemakers	Quote focuses on education focused on moral acceptance rather than the genocide
Combatting Denial	The denial of genocide is an affront to the victims. It aims to deny the right or remembrance to those who died. I consider it important to take specific steps and a structured approach to prevent genocide. This includes promotion of education and remembrance as key tool against dental of past crimes, criminal accountability and adjudication of criminal allegations, trust building and reconciliation	Quote discusses harms and dangers of genocide denial
Community	“We are going the extra mile instead of bringing people to be trained we find them in their communities and create centres to be near them, and have those very beneficiaries coming to the peace centre that will be hosting the training,” she said	Quote discusses creation of localized community groups for peace education programming
Critical Thinking	The teaching that is integrated in every subject, if well implemented, would also improve on how to equip students with crosscutting issues of critical thinking skills, freedom of expression and respect of the differences among their fellow students.	Quote discusses focus on critical thinking skills across the curriculum
Debate	Wilson Mugarura, the Head Teacher of King David Academy, says there is still slight inadequacy of the materials in regards to the implementation of the curriculum	Quote mentions a teacher who challenges the quality and availability of material for peace education, different from the government narrative
Difference	This collection of short vignettes will be valuable as an educational resource for students and educators. These stories highlight not only the diversity of experiences of genocide, but also many important lessons and insights into the consequences of discrimination, prejudice and ‘othering, as well as the power of activism and speaking up.	Quote discusses different opinions being considered in videos
Ethnicity	n/a	n/a
Preventing Genocide	Benoit Ryelandt, the Ambassador of Belgium to Rwanda said that since most of young adults were not born at the time of the Genocide against the Tutsi, “helping them learn from the past and understand how hatred and prejudice can lead to mass violence; step forward to build a stable and peaceful future generation”	Quote emphasises curriculum point of demonstrating how education can limit impacts of activities which. Lead to genocide
Nationalism	Since the end of the genocide, the government, led for 17 years by Paul Kagame, has pursued an official policy of unity and reconciliation, which emphasises Rwandan-ness rather than an affiliation as Hutu or Tutsi	Quote discusses emphasis on “Rwandaness"
Return to normal	n/a	n/a
Single narrative	When the 1994 Genocide against the Tutsi ended, its perpetrators never ceased to cause havoc but would from time to time make incursions in the country from the jungles of neighbouring DR Congo and elsewhere in the country.	Quote makes mention of the “Genocide Against the Tutsi” the official government narrative
Top-down	A donation of Aegis Trust, a nongoverment organisation campaigning to end genocide, the books were developed under a programme called Education for sustainable peace in Rwanda, and were handed over to Rwanda Education Board (REB) yesterday. Angelique Tusume, the Deputy Director General of REB, said at a handover ceremony on Tuesday that the books will contribute towards the country's efforts to fight genocide ideology.	Quote discusses dispersal of ministry made textbooks to be used in teaching
Tolerance	“There are times we have felt like restraining from talking about it because we thought we were going to hurt the children, because some of them come from families of victims, and others may be related to perpetrators. Sometimes, you yourself as a teacher may be afraid. that you will get emotional in front of your students “ he said. Such things hinder teachers from offering knowledge to their students in such critical fields, yet … teachers are important in cultivating understanding, empathy, community, and awareness of history that helps us build a better future”	Quote discusses how different groups may be impacted by the education programming differently

Results for the purpose of this study are presented here alongside discussions to illustrate reporting on resilience and discuss the implications for broader understandings of resilience in Rwanda simultaneously.

It should be noted that Rwanda does not have media freedom, and therefore sources published in Rwanda, which compose the majority of the data sources, read with a heavy bias, despite their role as ‘news’. Some examples in Table 2 below demonstrate this. This is important in that most international sources such as the BBC and The Washington Post simply republished from the pro-government Rwandan paper The New Times. International audiences may lack this context, and therefore the inclusion of these sources as ‘news’ is appropriate.

**Figure 1.**
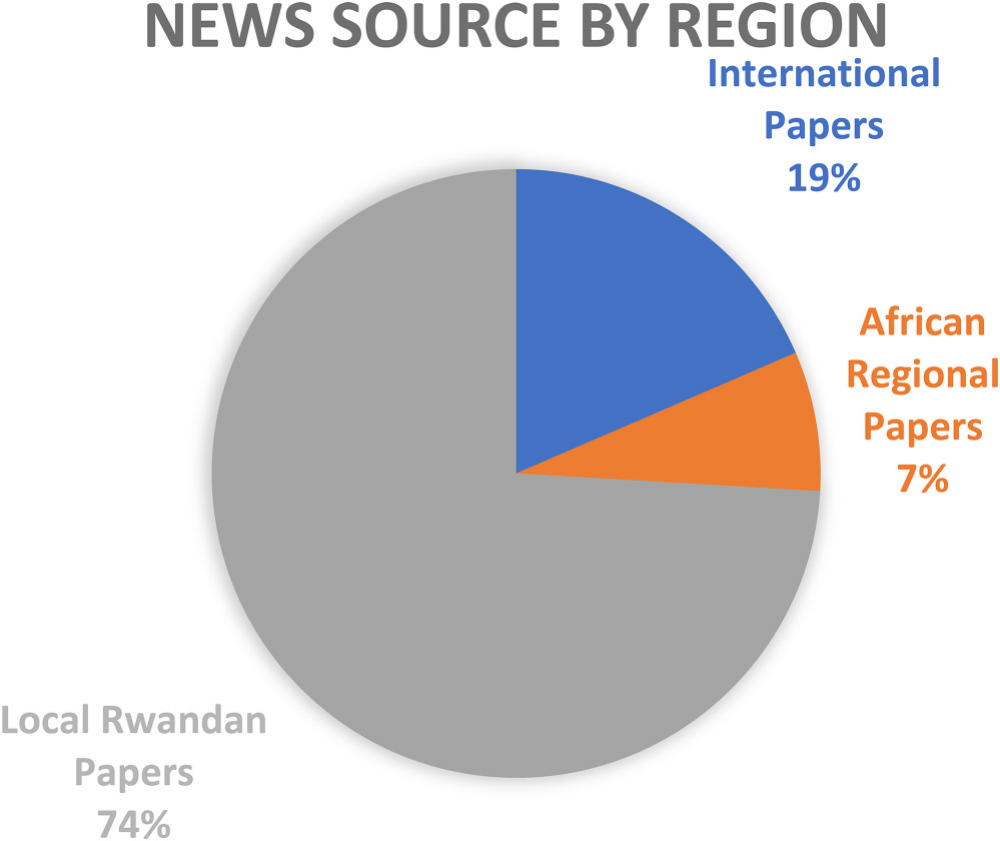
Percentage of news sources from various regions.

As is seen in Figure 1 below, the vast majority (74%, n  =  20) of articles collected were published in Rwanda.

International newspaper articles made up under one-fifth of all articles studied (n  =  5). While five papers were collected from African news sources, three of these articles were republished from Rwanda's *The New Times*, leaving only two articles written by African sources outside of Rwanda. The above data points to the fact that there is very little international attention paid to Rwanda's post genocide education system. This could be because of a lack of interest but could also be a symptom of Rwanda's donor darling status (Reyntjens, 2011). This also points to Rwanda having less criticism aimed at their own education system.

Table 3 below sorts resilience codes by Entman's frames.

**Table 3.** Resilience Codes Sorted by Frame.

Code	Frame				Total Codes
	1-define problems	2-diagnose causes	3-suggest remedies	4-make moral judgements	
Capital Interests	0	0	0	0	0
Citizenship Education	1	0	1	0	2
Nationalism	2	0	1	0	3
Return to normal	0	0	0	0	0
Single narrative	5	0	4	1	10
Top-down	1	0	2	0	3
Combatting Denial	6	2	3	0	11
Community	5	1	9	0	15
Critical Thinking	4	2	6	0	12
Debate	1	0	1	0	2
Difference	2	0	2	0	4
Ethnicity	0	0	0	0	0
Preventing Genocide	4	0	2	0	6
Tolerance	1	0	1	0	2
Total Frames	32	5	32	1	

**Figure 2.**
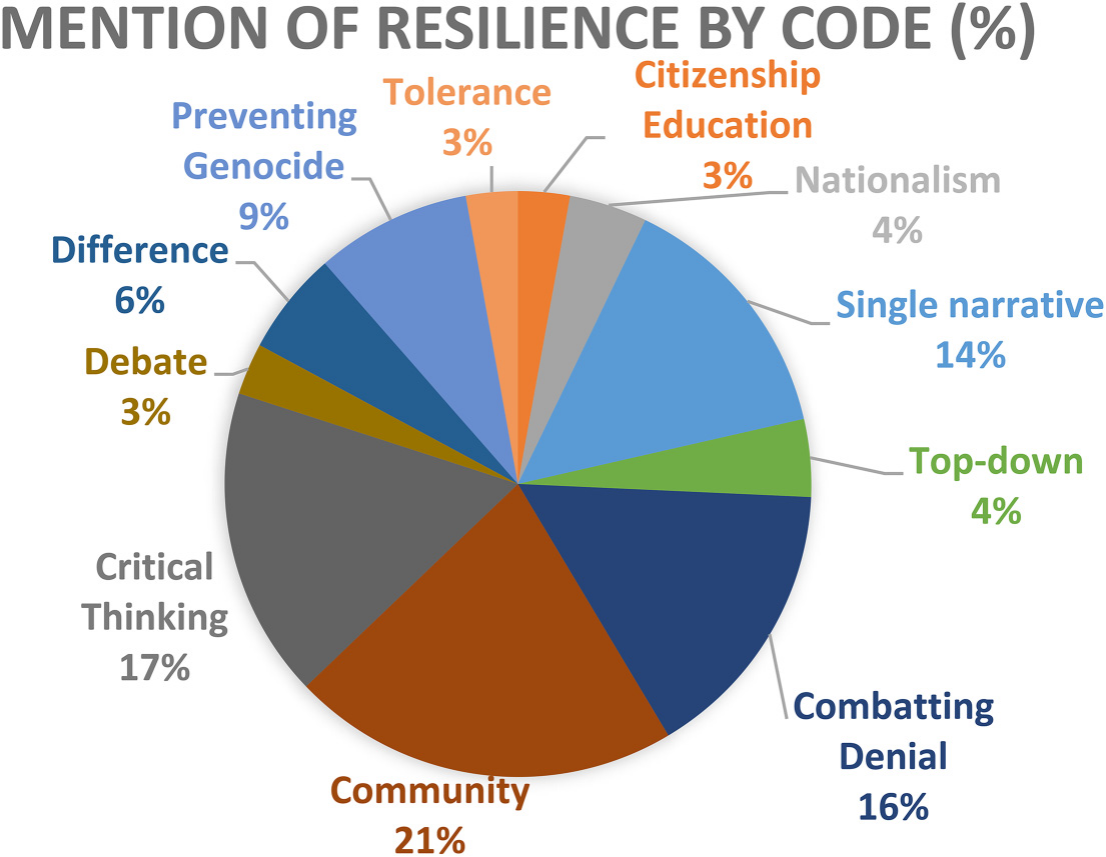
Total types of resilience code.

Figure 2 below provides a visualization of the percentage of the occurrence of the resilience codes. The three most popular codes are combatting denial, community and critical thinking, which make up over half of the total codes.

Given Rwanda's education system having a notably mixed approach to peace education, such variety in codes is perhaps unsurprising. However, the dominant presence of three resilient strategies requires greater discussion in the context of resilience. In the context of the broader education initiatives, it is important to note that community development and inclusion is a strong potential tool for resilience development (Rogers, 2013; Ungar, 2018). Moving into the broader community, using a participatory approach was essential to ensure community engagement. Given the challenges Rwanda has had getting some community members to participate in peace education initiatives in public spaces due to lack of trust (King et al., 2010), such a focus is of the utmost importance if Rwanda is to be successful over the long haul. The focus on community-based approaches also serves to illustrate that there is some recognition of the need for developing relationships as a form of education. This is important in that community-based approaches allow for a greater openness and space for difference (King et al., 2010). While this community approach is centred on the government programming it opens the door for individual stories to be told which will be beneficial. Not only does community development support greater engagement and understanding but it also provides the opportunity for engagement, where even though the burden is on the development of resilient individuals, there is a focus on an inclusive process. Such community promotion can be resisted by individuals but leaves space to push back against the neoliberal understanding of resilience as an inherently individual process.

Further, the discussion of combatting denial ultimately demonstrates the government taking onus to prevent harmful ideas. Given Rwanda's past, the nature of denial is harmful to both individuals and communities. This approach again removes some of the aspect of individualism in resilience and creates a role for greater intervention in the promotion of a supportive and diverse resilience.

The last of the most common approaches is the development of critical thinking which is presented as giving individuals the ability to think independently, so as to prevent future genocidal action. Given that peace education programs can often devolve into nationalist history, the focus on training of students to be critical thinkers serves an important role in reconstruction in Rwanda (Skårås, 2021). However, such an approach puts the responsibility to prevent genocide, as well as the blame for allowing genocide, on the individuals. The development of critical thinking skills is also contingent on the fear that another conflict is inevitable unless constantly fought against. While combatting the ideas that led to genocide is crucial, this downloading of the responsibility to citizens makes individuals the ones to blame if they do not achieve a specific standard. While this strategy walks a fine line, it is exclusively presented in news sources as a positive approach to resilience development.

The fact that all but one code a did not make a moral judgement of the approaches being used is significant in that there is limited potential for understanding how the education programs are presented.

This uncritical presentation points to a lack of criticism surrounding Rwanda's education system. As both the international and the African regional papers made up a small sample of the total documents collected, it is obvious that much of the discrepancy comes from Rwanda not being overly critical of its own approach to education. Given that Rwanda does not have an open and free media, the lack of criticism may be expected (Mudge, 2021). However, the fact that all but one code a did not make a moral judgement of the approaches being used is significant in that there is limited potential for understanding how the education programs are presented. This might also reflect the fact that there are more strategies being used which support positive resilience in Rwanda's education system, however, the lack of critical interpretation leaves it so there is limited critical understanding. This lack of criticism is unsurprising given that Rwanda receives lots of international aid (Edwards et al., 2018) resulting in Rwanda's donor darling status (Reyntjens, 2011).

The frame diagnosing causes is only seen in the three most common codes, combatting denial, community and critical thinking, which are all presented positively. Interestingly, however, the frame of suggesting remedies is present almost seven times as frequently. Given the often-uncritical presentation of the events in these cases, mentions of suggesting remedies are often a description of the programming being discussed alongside interviews or anecdotes. This could result in suggesting remedies which are not aligned with causes requiring fixing. Such an approach is dangerous by potentially promoting top-down resilience while ignoring community voices. This risks missing the most vulnerable groups. It could be that rather than being malicious, actions which have remedies suggested before diagnosing the problem are more likely to result in approaches which embody negative resilience.

In the discussion of approaches to resilience which have the greatest potential to cause harm, mentions of the single-narrative are the most common, with seven mentions. This should not be surprising and confirms past research on Rwanda being focused on the single-narrative ‘genocide against the Tutsi’ (McLean Hilker, 2011). The fact that the single-narrative approach is so common despite international recognition that this approach sews separatism and intolerance (Skårås, 2021) is incredibly troubling. Further, its uncritical presentation, with no moral judgement framing serves to illustrate that this approach is accepted and normalized internationally.

The one notable absent code is ethnicity in education, which is not surprising given the ‘One Rwanda’ mantra. (Thomson, 2011), but has the potential to be harmful (King & Samii, 2020). In the context of resilience focused education, such an exclusionary practice limits the ability of all to feel welcome and connect with their own heritage.

Taken together, the above results present an optimistic but maybe not positive understanding of the presentation of ESPR, that is headed in the right direction, although maybe not there yet. Given that there is heavy focus on the single narrative perspective, there are certainly challenges facing ESPR and peace education in Rwanda more broadly as they seek to develop a resilient and sustainable peace. However, this must be viewed in the context of the fact that Rwanda is not even 30 years removed from the genocide. This is not to pardon Rwanda's human right abuses, but rather to note that the peace that has reigned since 1994 has at the very least, been resilient in the short term. As such, there must be recognition of the fact that even with these challenges, the news media has focused largely on the positive aspects of resilience development which are being pursued, including community, critical thinking and combatting denial. All of this is to say that Rwanda’s approach to peace education is presented as, and often does include positive approaches to resilience, even if the delivery is imperfect.

Rwanda’s approach to peace education is presented as, and often does include positive approaches to resilience, even if the delivery is imperfect.

## Conclusion

This research evaluated the media portrayal of peace education programming as sparked by the ESPR initiative. Findings serve to demonstrate that there is very limited international news focus on education in Rwanda. While portrayals of education in Rwanda point largely to approaches which may support the positive approaches to resiliency, these findings serve to confirm past research on the general uncritical nature of media in Rwanda, but also support the notion that in the short time since the conclusion of Rwandan genocide, their education system presents as largely positive, though not perfect approach to resilience in peace education. This understanding of the portrayal in news media provides unique insight into the wider literature on peace education in Rwanda, which has seen significantly different findings (Basabose & Habyarimana, 2019; Thomson, 2011) The inclusion of the broader community in resilience focused peace education provides strong potential for long-lasting peace given the allowance of greater openness and difference (King et al., 2010; Rogers, 2013; Ungar, 2018). Recognizing the inherent challenges with resilience integration in peace education (Quaynor, 2012; Zembylas, 2016), this understanding of media portrayals of resiliency allows for a stronger understanding of how efforts in education to develop resilience are understood and disseminated, which is crucial to shaping public perception (Fryberg et al., 2012; Kelly, 2011). As we move forward, supporting not just resilience, but developing strategies of resilience focused peace education for the most vulnerable will be increasingly necessary. Understanding its media portrayal provides a unique insight into the perceived success and challenges of the programming.

This research also contributes to theories in the broader context of resilience in education by not only providing a case study of how resilience in peace education is portrayed in news media, but by providing a framework for how resilience in education systems may be analyzed. Where past work has looked at the implications of various approaches to peace education (King et al., 2010; Skårås, 2021), this research reprioritizes understandings of resilience in the success and failure of peace education (Skårås, 2021; Salomon, 2011; Ungar, 2018). This is to say that resilience in peace education can impact different groups differently, and the way that it is framed has implications for the presentation of its success. This research points to recommendations that diagnosing causes is essential in the implementation of peace education which does not inadvertantly prioritize negative resilience approaches. It also points policy makers to the need for more inclusive and balance histories to be prioritized in the development of peace education programming.

The main limitation facing this research is the isolated context of Rwanda. In focusing in on one small nation, the understanding of resilience in education may not necessarily be easily translatable to other areas, given the context dependence of resiliency. Further, the codes being dependent on international best practices may provide strategies which may be applicable more broadly, but may not necessarily be successful in Rwanda. Therefore, it is so important to recognize Rwanda's relative newness as a post-conflict nation and to recognize that resilience development may require time to adhere to all international best practices. Another major limitation comes in the fact that all sources reviewed were in English, thereby limiting the sample pool for articles published on other languages, including the local Kinyrwandan, however, this is mitigated by the strong presence of English language reporting in the region. Lastly, future research would benefit from including multiple coders to ensure interrater reliability in coding.
